# Immunogenicity of subcutaneous TNF inhibitors and its clinical significance in real-life setting in patients with spondyloarthritis

**DOI:** 10.1007/s00296-021-04955-8

**Published:** 2021-08-06

**Authors:** J. Hiltunen, P. Parmanne, T. Sokka, T. Lamberg, P. Isomäki, O. Kaipiainen-Seppänen, R. Peltomaa, T. Uutela, L. Pirilä, K. Taimen, M. J. Kauppi, T. Yli-Kerttula, R. Tuompo, H. Relas, S. Kortelainen, K. Paalanen, J. Asikainen, P. Ekman, A. Santisteban, K.-L. Vidqvist, K. Tadesse, M. Romu, J. Borodina, P. Elfving, H. Valleala, M. Leirisalo-Repo, V. Rantalaiho, H. Kautiainen, T. S. Jokiranta, K. K. Eklund, for the FinADASpA Study Group, Arto Kokko, Aulikki Kononoff, Elina Savolainen, Julia Barantseva, Antti Puolitaival, Tuomas Rannio, llpo Koskivirta, Johanna Paltta, Maija Puurtinen-Vilkki, Markku Mali, Jarno Rutanen

**Affiliations:** 1 Department of Rheumatology, Helsinki University and Helsinki University Hospital, Haartmaninkatu 4, P. O. Box 372, 00029 HUS Helsinki, Finland; 2 Department of Rheumatology, Jyväskylä Central Hospital, Jyväskylä, Finland; 3 University of Eastern Finland, Kuopio, Finland; 4 United Medix Laboratories, Helsinki, Finland; 5 Department of Rheumatology, Tampere University Hospital, Tampere, Finland; 6 Department of Rheumatology, Kuopio University Hospital, Kuopio, Finland; 7 Department of Rheumatology, Central Hospital of Lapland, Rovaniemi, Finland; 8 Department of Rheumatology, Turku University Hospital, Turku, Finland; 9 Department of Rheumatology, Päijät-Häme Central Hospital, Lahti, Finland; 10 University of Tampere, Tampere, Finland; 11 Department of Rheumatology, Satakunta Central Hospital, Rauma, Finland; 12 Medcare Foundation, Äänekoski, Finland; 13 SYNLAB Finland, Helsinki, Finland; 14 Translational Immunology Research Program, Helsinki University and Orton Research Foundation, Orton Hospital, Helsinki, Finland; 15 Department of Rheumatology, Mikkeli Central Hospital, Mikkeli, Finland

**Keywords:** Spondyloarthritis, Ankylosing spondylitis, Biological therapy, Anti-drug antibodies, Disease activity, Therapeutic drug monitoring

## Abstract

**Key messages:**

Considerable proportion of patients with SpA have been immunized to the subcutaneous anti-TNF drug they are using. Concomitant use of MTX protects from immunization, whereas SASP does not. Patients with SpA using subcutaneous anti-TNF drugs can benefit from monitoring of the drug trough levels.

**Abstract:**

Immunization to biological drugs can lead to decreased efficacy and increased risk of adverse effects. The objective of this cross-sectional study was to assess the extent and significance of immunization to subcutaneous tumor necrosis factor (TNF) inhibitors in axial spondyloarthritis (axSpA) patients in real-life setting. A serum sample was taken 1–2 days before the next drug injection. Drug trough concentrations, anti-drug antibodies (ADAb) and TNF-blocking capacity were measured in 273 patients with axSpA using subcutaneous anti-TNF drugs. The clinical activity of SpA was assessed using the Bath AS Disease Activity Index (BASDAI) and the Maastricht AS Entheses Score (MASES). ADAb were found in 11% of the 273 patients: in 21/99 (21%) of patients who used adalimumab, in 0/83 (0%) of those who used etanercept, in 2/79 (3%) of those who used golimumab and in 6/12 (50%) of those who used certolizumab pegol. Use of methotrexate reduced the risk of formation of ADAb, whereas sulfasalazine did not. Presence of ADAb resulted in decreased drug concentration and reduced TNF-blocking capacity. However, low levels of ADAb had no effect on TNF-blocking capacity and did not correlate with disease activity. The drug trough levels were below the consensus target level in 36% of the patients. High BMI correlated with low drug trough concentration. Patients with low drug trough levels had higher disease activity. The presence of anti-drug antibodies was associated with reduced drug trough levels, and the patients with low drug trough levels had higher disease activity. The drug trough levels were below target level in significant proportion of patients and, thus, measuring the drug concentration and ADAb could help to optimize the treatment in SpA patients.

**Supplementary Information:**

The online version contains supplementary material available at 10.1007/s00296-021-04955-8.

## Background

Development of anti-drug-antibodies (ADAb) to biological drugs can lead to low serum drug levels, reduced drug survival, loss of therapeutic response, and adverse events, such as infusion reactions. Immunogenicity of biological therapies has been studied extensively in rheumatoid arthritis (RA), but clearly less is known about the role of immunogenicity in the treatment of patients with spondyloarthritis (SpA) and ankylosing spondylitis (AS). In some studies, the development of ADAb has resulted in reduced efficacy, whereas in other studies no correlation between the presence of ADAb and disease activity has been observed. Anti-infliximab antibodies have been observed in 15–29% of AS patients [1] and SpA patients [2–4]. Development of anti-infliximab antibodies was associated with higher disease activity, lower infliximab trough levels and higher rate of infusion related reactions [1, 3, 4]. Anti-adalimumab antibodies have been observed in approximately 30% of AS and axSpA patients, and they have been associated with lower or undetectable serum drug levels and lower rate of response [5–7]. However, no association between ADAb against adalimumab and the disease activity was observed in patients with peripheral SpA [8], AS [9] or, according to a meta-analysis, AS or other SpA [10]. Immunogenicity of etanercept is negligible in SpA [11, 12] and AS patients [13]. Immunogenicity of golimumab has been reported to be low in patients with AS [14, 15] and SpA [16], and the presence of antibodies against golimumab has not been associated with lower clinical response [15, 16]. Prevalence of anti-certolizumab pegol ADAb has been reported to be low in patients with SpA [17] but relatively high in RA patients [18].

In addition to the presence of ADAb, also low drug trough level has been shown to result in lower clinical response in RA [19–24], but it is not clear whether this is true also in SpA.

Concomitant use of conventional synthetic disease-modifying antirheumatic drugs (csDMARD) has been shown to reduce the immunogenicity of biological drugs in RA, Crohn’s disease, or juvenile idiopathic arthritis patients using infliximab or adalimumab [25, 26]. Furthermore, concomitant use of csDMARD leads to better response in RA patients treated with tumor necrosis factor (TNF) inhibitors [27]. According to the recent international guidelines, csDMARD are not recommended for the treatment of axial SpA [28]. However, in practice, sulfasalazine (SASP) or methotrexate (MTX) are often used as monotherapy or in combination with biological DMARD. It is not known whether MTX can prevent the formation of ADAb in patients with SpA and the evidence of the benefit of concomitant use of csDMARD in SpA is somewhat conflicting [3, 4, 7, 28–33].

The aim of this study was to determine the extent and significance of immunization to anti-TNF drugs in real-life setting in unselected SpA patients with axial disease, and to assess the contribution of factors such as body mass index (BMI) and concomitant use of csDMARD on the immunogenicity.

## Materials and methods

### Patients

A total of 313 patients with SpA were enrolled to this national multicenter cross-sectional clinical study. Patients were recruited from 8 Finnish rheumatological outpatient clinics. The inclusion criteria were fulfillment of the classification criteria either by the 1984 New York criteria for AS or by the ASAS criteria for axial SpA (axSpA), and treatment with subcutaneous anti-TNF therapy (adalimumab, etanercept, golimumab or certolizumab pegol).

The exclusion criteria included diagnosis of psoriasis, psoriatic arthritis, or inflammatory bowel disease, inability to comprehend the consent form, concomitant csDMARD medication other than MTX or SASP, or glucocorticoid use of higher than 10 mg daily dose of prednisone or equivalent.

### Methods

The patients were recruited to the study during their normal visit to the rheumatology clinic during 2014–2015. Patients who were treated with subcutaneous anti-TNF agents, were recruited from 8 Finnish rheumatological outpatient clinics. The disease activity, function and enthesitis score were assessed during the visit by determination of Bath AS Disease Activity Index (BASDAI) [34], Maastricht AS Enthesis Score (MASES) [35] and Bath Ankylosing Spondylitis Functional Index (BASFI) [36]. BASDAI and BASFI are patient-reported measurements of the disease activity and the level of functional impairment, respectively, on a visual scale. MASES is based on clinical evaluation of tenderness in 13 enthesial sites. Clinical evaluation including Schober’s test and occipital-wall measurement, BMI, C-reactive protein (CRP) and erythrocyte sedimentation rate (ESR) were measured. The blood sample for drug trough concentration and ADAb was taken within 48 h before the next injection of the drug.

The serum samples were coded and stored at − 70 °C before analysis. Trough concentration of the anti-TNF drugs was measured with commercial capture-type enzyme-linked immunosorbent assay (Promonitor ELISA, Progenika Biopharma) in a clinical laboratory (United Medix Laboratories) following the accreditation standards for clinical diagnostics (EN ISO/IEC 17025 and EN ISO 15189). The level of ADAb were measured with radioimmunoassay (Sanquin laboratories, Amsterdam, The Netherlands). TNF-blocking capacity (RGA index) reflects the capacity of patient’s serum to neutralize further added TNF to the serum, and thus reflects the remaining capacity of the anti-TNF drug in the patient’s blood to neutralize TNF. The TNF-blocking capacity of the sera was assessed using a cell-based reporter gene assay (RGA) set up in United Medix Laboratories (Helsinki, Finland) [37]. The RGA index used in this study has been obtained from samples diluted 1:75 using the equation RGA index = 1/RFU (relative fluorescence units).

ADAb values ≥ 12 AU/mL were considered positive. Drug trough levels < 0.1 µg/mL were considered undetectable, and trough levels < 0.5 µg/mL were considered very low. The drug trough level was considered to be in the reference range if the level was 5–10 µg/mL for adalimumab, and thus low if the level was < 5 µg/mL [20, 38]. The drug trough level was considered to be low if the level was < 1242 µg/mL for etanercept [21], < 1.4 µg/mL for golimumab [22, 39], and < 9 µg/mL for certolizumab pegol [18]. Confirmed upper limit of the target drug level is not available for etanercept, golimumab and certolizumab pegol. Very low drug trough level was defined as being as low or lower than the lowest 1% of drug concentration results from samples in which no ADAb were detected. Low drug trough level was defined as being between 1 and 15% of drug concentration results from samples without ADAb.

### Statistical methods

The descriptive statistics are presented as means with SDs, as medians with IQR or as counts with percentages. Statistical comparisons between the groups were made using the *t* test, chi-square test, or analysis of variance. In the case of violation of the assumptions (non-normality), a bootstrap-type test was used. To determine characteristics associated with detected ADAb, multivariate logistic regression model was used. Correlation coefficients were calculated by the Spearman method, using Sidak-adjusted probabilities. The normality of the variables was tested by using the Shapiro–Wilk *W* test. All analyses were performed using STATA software (version 15.0), StataCorp, LP, Texas, USA.

The study protocol was approved by the Ethical Review Board of the Joint Authority for the Hospital District of Helsinki and Uusimaa (108/13/03/01/2014).

## Results

### Characteristics of the patient cohort

Of the 313 patients who gave their consent to the study, 273 gave a blood sample, and were included into the study. Of these patients 180 (66%) were male, 99 (36%) used adalimumab, 83 (30%) etanercept, 79 (29%) golimumab, and 12 (4%) certolizumab pegol. Of the patients 157 (58%) were using their first biologic drug, and of them 72% were male. The first biologic drug was used by 56 (57%) of the patients in adalimumab group, 50 (60%) in etanercept group, 43 (54%) in golimumab group and 8 (67%) in certolizumab pegol group. The characteristics of the patient cohort are presented in Table 1.

**Table 1:** Characteristics of the patient cohort

	Adalimumab *N* = 99	Etanercept *N* = 83	Golimumab *N* = 79	Certolizumab pegol *N* = 12	*p* value
Number of men, *n* (%)	60 (61)	53 (64)	59 (75)	8 (67)	0.25
Age, mean (SD)	46 (11)	44 (12)	42 (11)	47 (13)	0.12
BMI	26.1 (4.6)	26.2 (4.5)	26.8 (4.7)	25.6 (3.9)	0.74
Disease duration (years), mean (SD)	15.8 (10.3)	14.8 (10.1)	10.9 (9.4)	8.8 (6.9)	< 0.001
BASDAI, mean (SD)	1.7 (1.8)	2.1 (1.8)	2.0 (1.7)	1.2 (1.2)	0.12
MASES, mean (SD)	0.4 (1.4)	0.4 (1.4)	0.4 (1.3)	0.1 (0.3)	0.057
BASFI, mean (SD)	1.5 (2.0)	2.0 (2.0)	1.6 (1.5)	1.2 (1.0)	0.24
ESR, mean (SD)	8 (8)	9 (9)	6 (6)	5 (6)	0.064
CRP, mean (SD)	3 (4)	4 (5)	3 (3)	3 (4)	0.53
HLA-B27, *n* (%)	87 (94)	74 (95)	69 (90)	12 (100)	0.58
Treatment duration with current medicine (months) median (IQR), range	46 (22.71) [1–126]	46 (18.71) [2–131]	17 (9.33) [2–101]	7 (3.10) [3–23]	< 0.001
Duration of all biologic treatment (months), median (IQR), range	57 (35.104) [3, 172]	55 (31.91) [2–177]	25 (16.55) [2–210]	8 (3.60) [3–110]	< 0.001
DMARD, *n* (%)					
None	44 (44)	42 (51)	36 (46)	3 (25)	0.40
Methotrexate	41 (41)	25 (30)	32 (41)	6 (50)	0.30
Sulfasalazine	21 (21)	22 (27)	17 (22)	5 (42)	0.39
Leflunomide	1 (1)	0 (0)	0 (0)	0 (0)	0.99
Peroral glucocorticoid, *n* (%)	5 (5)	2 (2)	3 (4)	3 (25)	0.039
Previous biologic treatment					
Number, *n* (%)					0.69
0	56 (57)	50 (60)	43 (54)	8 (67)	
1	36 (36)	24 (29)	21 (27)	2 (17)	
2	6 (6)	5 (6)	11 (14)	2 (17)	
3–4	1 (1)	4 (5)	4 (5)	0 (0)	
Previous biologic treatment					
Certolizumab pegol	2 (2)	3 (4)	2 (3)	.	
Etanercept	29 (29)	.	17 (22)	1 (8)	
Adalimumab	.	25 (30)	22 (28)	2 (17)	
Golimumab	5 (5)	7 (8)	.	2 (17)	
Infliximab	12 (12)	6 (7)	14 (18)	1 (8)	

*BMI* body mass index, *BASDAI* Bath Ankylosing Spondylitis Disease Activity Index, *MASES* Maastricht Enthesitis Score, *BASFI* Bath Ankylosing Spondylitis Functional Index, *ESR* erythrocyte sedimentation rate, *CRP* C-reactive protein, *HLA-B27* human leukocyte antigen B27, *DMARD* disease-modifying antirheumatic drug

Mean duration of disease was 10 years for those who used their first biologic drug and 15 years for those who had used one or more biologic drugs previously (*p* < 0.001). As expected the mean age was higher in the group who had had prior biologic treatment (47 vs. 43 years, *p* = 0.003). The two groups did not differ with respect to weight, BMI, mean result of Schober’s test, occipital-wall test, or CRP, nor with respect to the use of concomitant csDMARD or glucocorticoid therapy. ESR was lower in those who used their first biologic drug (*p* = 0.015) (data not shown).

### The prevalence of anti-drug antibodies

ADAb were found in 29 (11%) of the 273 patients. ADAb were observed in 21 of the 99 (21%) patients who used adalimumab, in 0 of 83 (0%) of those who used etanercept, in two of the 79 (3%) of those who used golimumab and in six of the 12 (50%) of those who used certolizumab pegol (*p* < 0.001). There was no difference in the prevalence of ADAb between those patients who used their first biologic drug, as compared to those who had used previously one or more biologic drugs.

### Demographic and clinical factors affecting drug trough concentration, presence of ADAb and the TNF-blocking capacity

Higher BMI, MASES and ESR correlated with lower drug trough concentration. Older age correlated with higher drug trough concentration and better TNF-blocking capacity (higher RGA index). Disease duration and the length of biologic treatment correlated with higher RGA index. The results are presented in Table 2.

**Table 2:** Correlation coefficient between drug trough concentration, detected ADAb and TNF alpha blocking capacity (RGA index) to demographics and clinical data

	Drug trough level *r* (95% CI)	ADAb (95% CI)	TNF-blocking capacity (95% CI)
Sex	0.03 (− 0.09 to 0.15)	− 0.12 (− 0.24 to − 0.01)	− 0.03 (− 0.14 to 0.09)
Age	0.20 (0.08 to 0.31)**	− 0.08 (− 0.20 to 0.04)	0.19 (0.08 to 0.30)*
BMI	− 0.22 (− 0.33 to − 0.10)**	0.12 (− 0.00 to 0.23)	− 0.15 (− 0.26 to − 0.03)
Disease duration	0.14 (0.02 to 0.26)	− 0.03 (− 0.15 to 0.09)	0.20 (0.09 to 0.32)**
Duration of use of biological drugs	0.11 (− 0.01 to 0.22)	− 0.11 (− 0.22 to 0.01)	0.19 (0.08 to 0.31)*
ESR	− 0.17 (− 0.28 to − 0.05)*	0.12 (− 0.00 to 0.23)	− 0.02 (− 0.14 to 0.10)
CRP	− 0.15 (− 0.26 to − 0.03)	0.08 (− 0.04 to 0.20)	− 0.10 (− 0.22 to 0.02)
BASDAI	− 0.06 (− 0.18 to 0.06)	− 0.11 (− 0.22 to 0.01)	− 0.06 (− 0.17 to 0.06)
MASES	− 0.18 (− 0.30 to − 0.07)*	0.08 (− 0.04 to 0.20)	− 0.14 (− 0.25 to − 0.02)
BASFI	− 0.06 (− 0.18 to 0.06)	− 0.08 (− 0.20 to 0.04)	− 0.02 (− 0.14 to 0.10)

*BMI* body mass index, *ESR* erythrocyte sedimentation rate, *CRP* C-reactive protein, *BASDAI* Bath Ankylosing Spondylitis Disease Activity Index, *MASES* Maastricht Enteritis Score, *BASFI* Bath Ankylosing Spondylitis Functional Index

*p < 0.05, **p < 0.01, ***p < 0.001; statistical significance calculated using Sidak-adjusted probabilities

The patients using adalimumab were analyzed also as a separate group because of the low number of patients in the certolizumab pegol group and low rate of ADAb positivity in the etanercept and golimumab groups. Increased risk of immunization in adalimumab users was associated with longer duration of the disease and higher BMI, whereas younger age and the length of all biologic treatment were associated with decreased risk of ADAb positivity. HLA-B27 status or gender was not associated with the ADAb prevalence. The results are presented in Table 3.

**Table 3:** Multivariate logistic regression model for the detected ADAb in patients using adalimumab

	OR (95% CI)	*p* value
Gender	0.77 (0.22–2.72)	0.68
Age	0.87 (0.79–0.95)	**0.002**
BMI	1.16 (1.01–1.32)	**0.033**
Disease duration	1.13 (1.03–1.26)	**0.015**
Total time of use of biological drug	0.98 (0.96–1.00)	**0.040**
HLA-B27	3.02 (0.38–23.78)	0.29
csDMARD	0.21 (0.06–0.76)	**0.017**

*BMI* body mass index, *HLA-B27* human leukocyte antigen B27, *csDMARD* conventional synthetic disease-modifying antirheumatic drug

The bold values indicate significance

### Effect of concomitant medication on prevalence of anti-drug antibodies

The proportion of patients who used any csDMARD were 56%, 49%, 54%, and 75%, in patients using adalimumab, etanercept, golimumab, and certolizumab pegol, respectively. Concomitant glucocorticoid use was uncommon, except in patients using certolizumab pegol, of whom 25% used glucocorticoids. ADAb were detected in 12% of patients using concomitant MTX as compared to 28% of those who did not use MTX (*p* = 0.048: adjusted for gender, disease duration, age, BMI, HLA-B27, and the time of use of biological drug). SASP was not associated with immunization; there were 6 ADAb-positive vs. 59 ADAb-negative SASP-using patients, and 23 ADAb-positive vs. 185 ADAb-negative patients with no SASP (*p* = 0.68).

### Drug trough levels and effect of ADAb on trough levels and TNF-blocking capacity

The mean drug trough concentrations of different TNF inhibitors are presented in Supplementary Table 1. The mean drug trough levels were 8.1 mg/L, 1.6 μg/mL, 1.5 μg/mL and 30.3 μg/mL for adalimumab, etanercept, golimumab and certolizumab, respectively. The mean drug trough concentrations were in the target level in 138 (51%) of the patients. 99 patients (36%) had drug concentration lower than the target level and higher than the target drug concentration was detected in 36% of patients using adalimumab.

**Fig. 1:**
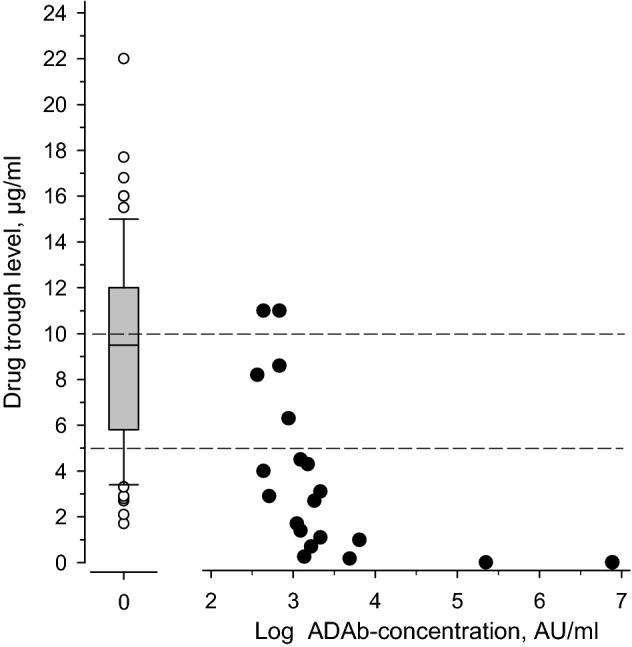
The association of ADAb concentration and the drug trough level in patients using adalimumab. Left, mean drug trough level of those without detectable ADAb. Right; black dots show the individual values of each patient with detected ADAb. The dotted line indicates the suggested therapeutic drug trough level of adalimumab

The effect of ADAb on drug trough levels in patients using adalimumab is depicted in Fig. 1. At low ADAb concentrations, no clear effect on the drug trough levels could be seen. However, at high ADAb concentrations, decreased drug trough levels are evident, and at the highest ADAb concentration, no measurable drug concentration could be detected.

**Fig. 2:**
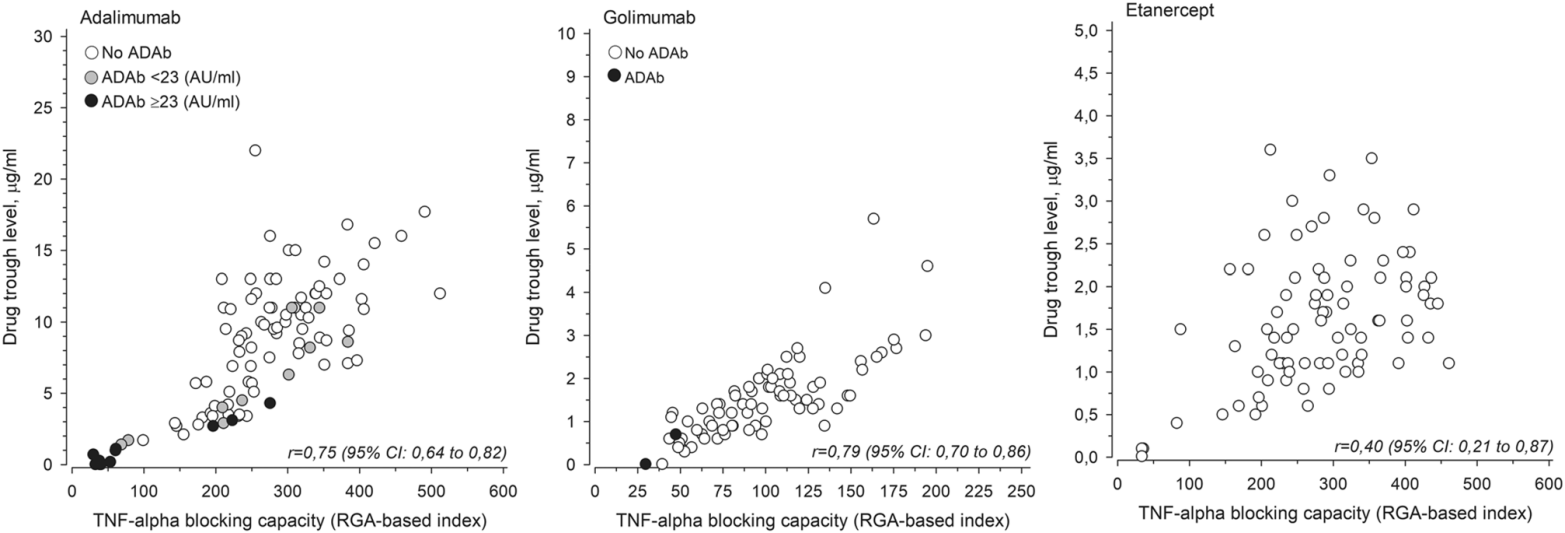
TNF-blocking capacity of adalimumab, golimumab and etanercept. Gray circles (adalimumab) represent patients with low level of ADAb (< 23 AU/mL) and black circles patients with high level of ADAb (> 23 AU/mL). In golimumab users, black circles represent patients who are positive for ADAb

TNF-blocking capacity reflects the ability of patients’ serum to neutralize TNF before next injection of anti-TNF drug. High TNF-blocking capacity (RGA index) means that the patient’s sera can still neutralize produced TNF. The TNF-blocking capacity of the patients using adalimumab is depicted as a function of drug trough level in Fig. 2. A clear correlation can be seen between the drug trough level of adalimumab and the TNF-blocking capacity of the patient’s serum (*r* = 0.75, 95% CI 0.64–0.82). In patients with high ADAb concentration (> 23 AU/mL), the TNF-blocking capacity is low, i.e., the patient’s serum has low or no ability to neutralize TNF. However, the presence of low concentrations of ADAb (< 23 AU/mL) did not significantly reduce the TNF-blocking capacity, i.e., many of those patients have significant TNF-blocking capacity left before the next injection of the drug. A clear correlation between the drug trough level and the TNF-blocking capacity could be observed also in case of golimumab (*r* = 0.79, 95% CI 0.77–0.86). Patients with positive ADAb in the golimumab group also had low TNF-blocking capacity (low RGA index). In case of etanercept, there was no clear correlation between drug trough concentration and the TNF-blocking capacity, as good TNF-blocking capacity could be seen also at relatively low drug concentrations.

### Low drug trough level but not the presence of anti-drug antibodies associates with high disease activity

**Fig. 3:**
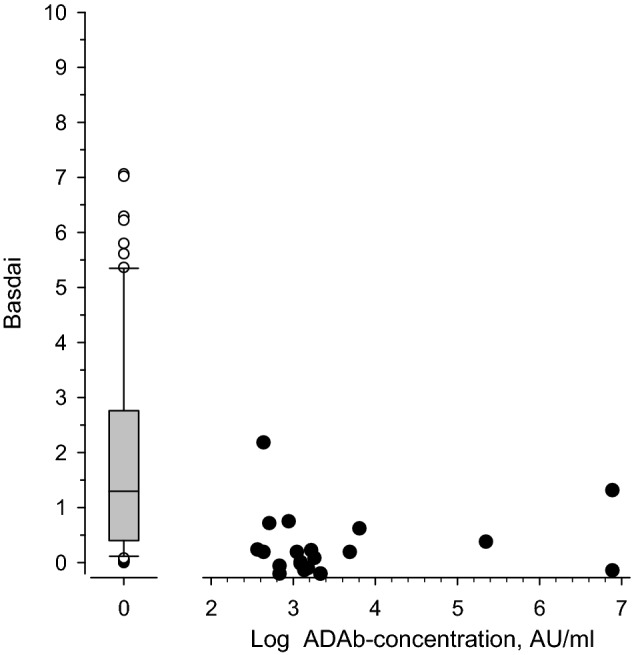
BASDAI values of AS patients using adalimumab. Left, the mean BASDAI values of patients with no ADAb. Right: The black dots show individual BASDAI values of ADAb-positive patients as a function of ADAb concentration

Significant association was found between low drug trough concentration and high ESR (*p* = 0.032) and high MASES value (*p* = 0.007) in the entire population. Low drug concentration was associated with high MASES also when patients using adalimumab were studied separately (*r* = − 0.23 (CI − 0.41 to – 0.03), *p* = 0.023). Also the RGA index correlated with MASES; *r* = − 0.25 (CI − 0.43 to − 0.05), *p* = 0.014. However, no significant correlation was found between drug trough level and BASDAI or between the presence of ADAb and BASDAI. In patients using any anti-TNF drug there was no statistically significant correlation between the presence of ADAb and BASDAI (*r* = − 0.18 (95% CI − 0.36 to 0.02), *p* = 0.080), BASFI (*r* = − 0.13 (95% CI − 0.32 to 0.07), *p* = 0.21), or MASES (*r* = 0.18 (95% CI − 0.02 to 0.36), *p* = 0.081). Figure 3 shows the BASDAI values in ADAb-negative and ADAb-positive adalimumab users. This reveals that many patients with no ADAb had very high BASDAI values and the number of patients with high level of ADAb was rather low, thus possibly explaining the lack of correlation between ADAb and BASDAI.

## Discussion

In this study, we show that significant proportion of SpA patients using subcutaneous anti-TNF drugs have developed ADAb against the drug they are using. The significance of immunization to biological drugs is not clear in SpA. Bornstein et al*.* found out that ADAb were detected in 15% of 39 SpA patients (4/14 on infliximab and 2/16 on adalimumab), of which all had undetectable drug trough levels. ADAb positivity was also associated with higher disease activity [12]. In another study on axSpA patients 27.5% of patients had anti-adalimumab antibodies. ADAb were associated with lower drug levels and higher clinical activity evaluated by BASDAI and ASDAS [40]. In a recent study by Ducourau et al., ADAb were detected in 36.4% of axSpA patients using adalimumab. Also in this study immunization was associated with lower drug levels and higher clinical activity [7]. These results are concordant with the previously published data showing that most ADAb against adalimumab are neutralizing [41]. In the present study, 11% of all users of subcutaneous anti-TNF drugs had developed ADAb. As in RA, the highest rate of immunization was observed in patients who used adalimumab (21%). This is in agreement with previous studies demonstrating ADAb against adalimumab in about 30% of RA [42] and AS patients [5–7, 43]. It seems that as in the earlier mentioned studies the anti-adalimumab antibodies were neutralizing since all the patients with very low drug trough level were ADAb positive [44]. We did not detect ADAb against etanercept, which is also in agreement with previous studies with patients with SpA [11, 12], AS [13, 43], and RA [23]. Of the patients using golimumab, 3% had ADAb, also in agreement with previous studies [14–16]. A total of 50% of certolizumab pegol users were positive for ADAb. However, most of those had drug trough level at the target level, and therefore, the antibodies may not be neutralizing, and thus the clinical significance of the ADAb remains unclear. The number of patients in certolizumab pegol group was also only 12, and therefore no definite conclusions can be made. In earlier studies, the rate of immunization has been lower in axSpA patients, and anti-certolizumab antibodies have been associated with lower drug levels yet the clinical significance remaining unclear [17, 45]. The number of biologic drugs used earlier did not correlate with immunization.

Patients with concomitant MTX had less frequently ADAb, as compared to those not using MTX, suggesting a protective effect of MTX on immunization also in patients with SpA. In contrast, SASP did not show protective effect against immunization. Higher BMI and longer duration of the disease correlated with the presence of ADAb, and younger age and long duration of biologic treatment correlated negatively with ADAb positivity in adalimumab users.

Lower adalimumab concentrations as well as lower clinical response has been observed in AS or axSpA patients with BMI > 30, as compared to those with BMI < 25 [46–48]. Theoretically, the lower adalimumab serum levels due to changes in volume distribution could predispose obese patients to immunization against adalimumab. However, in spite of the lower serum drug levels, no difference was observed in rate of immunization [46]. Obesity has been associated with lower TNF inhibitor levels also in RA patients [23] and lower clinical response in patients with rheumatic diseases using TNF inhibitors [49]. In the present study, higher BMI correlated with lower drug trough concentration. Higher BMI also correlated with ADAb positivity in adalimumab users.

The presence of ADAb correlated negatively with drug trough concentration in all the treatment groups in which ADAb were detected (adalimumab, golimumab, and certolizumab pegol). However, the presence of ADAb did not significantly correlate with disease activity. This is most likely explained by the fact that low levels of ADAb did not have significant effect on drug trough levels. As shown in Fig. 2 patients with low levels of ADAb had significant remaining TNF-blocking capacity, and only in those with high levels of ADAb the TNF-blocking capacity was low. As this study was cross-sectional, it is impossible to know whether the patients with low level of ADAb later developed higher ADAb levels or lost their treatment response. Low drug trough levels were associated with higher disease activity, as reflected by the association with higher ESR and higher MASES.

A correlation was observed between adalimumab and golimumab drug trough level and the TNF-blocking capacity expressed as RGA index. In case of etanercept, no clear correlation could be observed between the TNF-blocking capacity and the drug trough level, as the TNF-blocking capacity was good also at lower drug concentrations. The reason for this is not clear. Also, in earlier studies, the clinical relevance of drug level of etanercept seems to be somewhat controversial in patients with both AS [13, 50] and RA [23, 51].

Based on these results, we believe that measuring drug concentration and ADAb in SpA patients using TNF inhibitors with the exception of etanercept is useful both in patients with active disease, but also in those patients who seem to respond to therapy to avoid unnecessary treatment in those who have been immunized to the drug and have therefore unmeasurable drug levels. This is supported by a recent study by Pedersen et al. who showed that therapeutic drug monitoring of TNF inhibitors leads to better clinical response and cost management in the treatment of patients with rheumatic diseases [52]. A recent review concluded that measuring drug levels and ADAb in RA and SpA patients with biologic treatment might be useful both in case of poor response and longer clinical remission [53].

The study has several limitations. The aim of the study was to evaluate the extent and significance of immunization in unselected patients coming to regular outpatient visit. Our study is a cross-sectional study, and thus it does not allow the analysis of the kinetics of immunization to TNF alpha inhibitors or allow the follow up of the patients. Although the study population is relatively large the subgroups of patients using certolizumab pegol and ADAb-positive group of patients using golimumab are rather small, which makes it difficult to draw conclusions concerning immunization and its clinical relevance in these subgroups of patients.

## Conclusion

A significant proportion of patients had been immunized to the subcutaneous anti-TNF drug they were using. The presence of ADAb was associated with lower drug concentration and reduced TNF-blocking capacity. Low drug trough levels correlated with higher ESR and MASES. The use of MTX reduced the risk for immunization in patients with SpA, whereas SASP did not. There was no clear correlation between ADAb and the BASDAI, BASFI or MASES scores, likely because the low ADAb levels had no significant effect on TNF-blocking capacity. Thus the significance of finding of low ADAb level in patients is not clear. It is likely that patients with high drug antibody levels and low drug trough concentrations do not benefit from the drug they are using. Thus, measuring of drug trough level and ADAb may be useful to optimize treatment individually and achieve better cost management.

## Supplementary Information

Below is the link to the electronic supplementary material.

Supplementary file1 (DOCX 56 KB)

## Data Availability

All data of the study is available upon reasonable request.
