# Supplementary material for: Immunogenicity of subcutaneous TNF inhibitors and its clinical significance in real-life setting in patients with spondyloarthritis
Source: Rheumatol Int. 2021 Aug 6;42(6):1015–25. doi: 10.1007/s00296-021-04955-8 (PMC9124652; doi:10.1007/s00296-021-04955-8)
Supplement: Supplementary file 1 — Supplementary file1 (DOCX 56 KB) [file 296_2021_4955_MOESM1_ESM.docx]

**Supplementary Table 1.** The mean drug trough levels and proportion of patients with drug level lower or higher than target level

|  | Adalimumab | Etanercept | Golimumab | Certolizumab |  |
| --- | --- | --- | --- | --- | --- |
| Mean drug trough level  (target level) | 8,1 mg/l  (5-10 mg/l) | 1,6 ug/ml  (≥1,242 ug/ml) | 1,5 ug/ml  (≥1,4 µg/ml) | 30,3 ug/ml  (≥9 µg/ml) |  |
| Lower than target level, n (%) | 30/99 (30) * | 31/83 (37) | 37/79 (47) ** | 1/12 (8)***** | **^1^** |
| Very low drug trough level, n (%) | 5/99 (5) *** | 4/83 (5) | 6/79 (8) **** | 1/12 (8)***** |  |
| Higher than target level, n (%) | 36/99 (36) |  |  |  |  |

*****Of which 16/30 were ADAb positive, ** of which 2/37 were ADAb positive, *** of which all 5/5 were ADAb positive, ********of which 1/6 was ADAb positive, ********* of which 1/12 was ADAb positive, **^1^** p=0,024.
